# Modeling of Twin Screw Extrusion of Polymeric Materials

**DOI:** 10.3390/polym14020274

**Published:** 2022-01-10

**Authors:** Adrian Lewandowski, Krzysztof Wilczyński

**Affiliations:** Polymer Processing Department, Faculty of Mechanical and Industrial Engineering, Warsaw University of Technology, Narbutta 85, 02-524 Warsaw, Poland; adrian.lewandowski@pw.edu.pl

**Keywords:** polymers, twin screw extrusion, modeling

## Abstract

An issue of modeling of twin-screw extrusion of polymeric materials is reviewed. The paper is written in honor of Prof. James L. White who was a pioneer in studying this issue. A global approach to process modeling is presented which includes solid polymer transport, polymer plasticating, and the flow of molten polymer. The methodology of CFD modeling of twin-screw extrusion is presented as well as the examples of this modeling which show the details of the process. Optimization and scaling of twin-screw extrusion are also covered. And finally, the future prospects of developments and research of twin screw extrusion is discussed.

## 1. Introduction

Extrusion is the most mass technology in the polymer processing industry. It is widely used for production of profiles, e.g., sheets, pipes, films, and for specialty operations, e.g., compounding, granulating, etc.

Extruders can be classified as single screw, twin screw, and multi-screw machines. Special extruders can also be distinguished. Single screw machines may have smooth or grooved barrels and may be equipped with conventional or non-conventional screws with elements intensifying melting or mixing. Twin screw machines can be co-rotating or counter-rotating, with varying degrees of screw meshing. In the single screw extrusion mixing action is poor, and melting is slow. In the twin screw extrusion, mixing is much better and melting is faster. Extruders are usually fed with solid polymers (plasticating extruders), although they can also be fed with molten polymers (melt extruders). Extrusion can be performed with gravitational feeding (flood-fed extrusion) or with metering (starve-fed extrusion). Single screw extruders are usually flood fed, while twin screw extruders are starve fed.

At present, designing the polymer processing is aided by process simulations which allow predicting the process, i.e., to compute the output process parameters using the input parameters (material characteristics, machine geometry and operating conditions).

In the modeling of polymer processing, the models are generally deterministic, transport phenomena based, of distributed parameters or locally lumped parameters. For engineering calculations, the lumped parameter approach is generally sufficient. The main objective of engineering designs of extrusion is to predict the pressure and mean polymer temperature along the machine for a given screw/die geometry as a function of operating conditions. In these models, the screw channel is divided into short segments, where the input data come from the calculation in the previous segment, and the output data from the current segment are the input data for the next segment. In the segments, the local parameters are assumed to be constant. This locally lumped parameters concept is particularly useful when dealing with plasticating processes, such as extrusion, where in addition to the melt flow, solids transport and melting are modeled.

Computer modeling allows us to answer practical questions of polymer extrusion. It allows to study the impact of the input parameters variation on the material/process outputs and to show the trends on the material reaction to hundreds of potential input data configurations. It allows to get quickly the process map for identifying the requested process parameters, according to the targeted materials and the selected screw profile, that is, distribution of the thermo-mechanical results (temperature, pressure, viscosity, filling) along the screw, detailed energy balance of the process, residence time analysis, etc. The scale-up modules of the software allow the transferring of processes to machines with larger or smaller diameters. Starting from the model process, the geometry of the target design and the associated process parameters are calculated on the basis of transfer rules.

However, there are also some shortcomings in practical use. The important one is that the global models which describe the complete extrusion process including solid transport, polymer melting and melt flow are based on the relatively simple 1D or 2D models, and do not allow looking into details of the process.

Many important books have been written on polymer extrusion, e.g., by Fischer [1], Schenkel [2], Mc Kelvey [3], Fenner [4], Tadmor and Klein [5], Potente [6], Hensen et al. [7], White [8], White and Potente [9], Tadmor and Gogos [10], White and Kim [11], Vlachopoulos [12], Rauwendaal [13], Lafleur and Vergnes [14], Vlachopoulos and Polychronopoulos [15], Chung [16], Noriega and Rauwendaal [17], Kohlgrüber [18,19], Campbell and Spalding [20], and many others [21,22,23,24,25,26,27,28,29,30,31,32,33]. However, only a few of them have been devoted wholly to twin screw extrusion, namely the books of White on both co-rotating and counter-rotating extrusion [8,9,11] and Kohlgrüber on co-rotating extrusion [18,19].

This paper is written in honor of Prof. James L. White who was a pioneer in studying and modeling twin screw extrusion, both co-rotating and counter-rotating. A comprehensive approach to process modeling is presented which includes the solid polymer transport, the polymer plasticating, and the flow of molten polymer. These are based on strong experimentation. The software used for global simulation of twin screw extrusion is also reviewed, as well as CFD modeling of the process is presented with the modeling examples which show the details of the process, which are not available when using the global software. Optimization and scaling twin screw extrusion are also covered. And finally, the future prospects of developments and research of twin screw extrusion are discussed.

## 2. Twin Screw Extrusion

**Figure 1:**
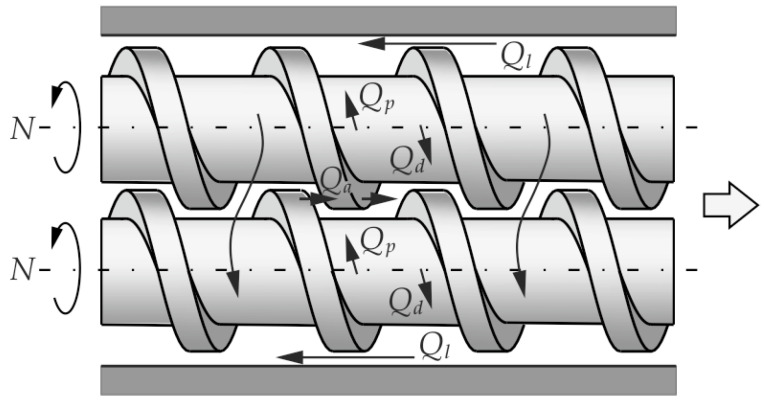
The polymer flow in the co-rotating twin screw extruder: Q_d_—drag flow, Q_p_—pressure flow, Q_a_—axial flow, Q_l_—leakage flow (adopted with permission from: Wilczyński, K. *Rheology in Polymer Processing. Modeling and Simulation*; Carl Hanser Verlag: Munich 2021 [34]).

**Figure 2:**
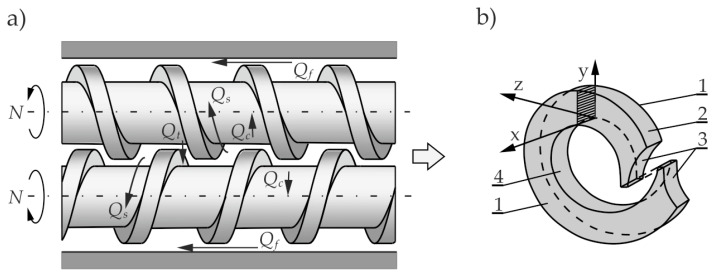
The polymer flow in the counter-rotating twin screw extruder: (**a**) leakage flows, Q_c_—calendering flow, Q_f_—flight flow, Q_t_—pressure (tetrahedral) flow, Q_s_—side flow, (**b**) C-shaped chamber, 1—side surface of the screw flight, 2—barrel surface, 3—front surface of the screw flight, 4—surface of the screw root (adopted with permission from: Wilczyński, K. *Rheology in Polymer Processing. Modeling and Simulation*; Carl Hanser Verlag: Munich 2021 [34]).

Twin screw extrusion is divided due to the direction of the screw’s rotation into: co-rotating extrusion, when the screws rotate in the same direction (Figure 1), and counter-rotating extrusion, when the screws rotate in the opposite direction (Figure 2). Twin screw extrusion can be performed with varying degrees of meshing. And so, it stands out: extruders with non-intermeshing screws, tangential or separated, and extruders with intermeshing screws, partially or closely intermeshing (with a special type of self-wiping screws). Three practically used, twin screw extrusion techniques can be distinguished: intermeshing co-rotating operations (including self-wiping), non-intermeshing tangential counter-rotating operations, and intermeshing counter-rotating operations.

Twin screw extruders are mostly used for processing of thermally sensitive polymers, e.g., PVC (polyvinyl chloride), and for specialty operations, e.g., compounding, polymer filling and reinforcing.

The mechanism of polymer flow in twin screw extruders differs from that one in single screw extruders and to a large extent depends on the meshing degree. In single screw machines, the polymer flow is of drag type, while in twin screw machines there is also a positive displacement mechanism which is dependent on the degree of screws meshing. It occurs most fully in the intermeshing counter-rotating extruders. In the non-intermeshing screws, this mechanism does not occur. Generally, the closer the meshing is, the transport mechanism is more different. Non-intermeshing extruders resemble basically single screw extruders and therefore do not have such good transportability as the closely intermeshing screws.

Another important difference is the polymer velocity distribution in the extruder. In twin screw machines, this distribution is complex and difficult to describe mathematically. However, this complicated nature of the flow causes that twin screw extruders have good mixing and devolatilizing abilities and are characterized by good heat exchange and fast polymer plasticating. The difficulty of describing such a flow, however, makes the twin screw extrusion theory less developed than the single screw process theory.

Twin screw machines have significant disadvantages alongside their advantages. They are expensive, structurally complex and difficult to manufacture.

### 2.1. Co-Rotating Twin Screw Extrusion

Co-rotating twin screw machines are mainly applied for compounding, e.g., polymer mixing, devolatilization, filling, reinforcing or reactive extrusion. The schematic of the co-rotating process is depicted in Figure 1.

In this process, the polymer flows from one screw to the other and moves in an open figure-eight pattern, along with the so-called twisted eight. There is a counter-rotating movement in the inter-screw gap, as a result of which high shear stresses are produced in this area. And therefore, this method is used in compounding.

Co-rotating twin screw extrusion is usually performed with metered feeding (starve feeding), which makes the pressure profile in the extruder not continuous, and it contains sections where the pressure is reduced to zero. In these areas, i.e., in areas where pressure is not produced, the feeders of various additives may be placed, e.g., for polymer filling, polymer reinforcing or modifying. In these areas, it is also possible to place the devices for devolatilization or removal of chemical reaction products during reactive extrusion.

In co-rotating machines, during each screw rotation, the polymer is transferred between the screws. The flow of polymer is dragged so that it resembles the flow in a single screw machine in this respect. However, when moving between the screws, the polymer travels a longer path and undergoes higher shearing.

The flow of polymer in co-rotating machines is the result of the movement of the screws relative to the barrel, as well as the pressure gradient in the machine. It is the pressure-drag flow which takes place in open channels between screws (alternately one and the other) and the barrel.

### 2.2. Counter-Rotating Twin Screw Extrusion

Counter-rotating twin screw machines are used mainly for extruding profiles from thermally sensitive materials, e.g., PVC (polyvinyl chloride). The schematic of the counter-rotating process is depicted in Figure 2.

Counter-rotating extruders, in comparison with single screw extruders, provide better feeding the extruder with polymer (especially in the case of polymers in the form of powder or polymers exhibiting slip properties), and provide a shorter and less varied residence time of the polymer particles in the extruder.

In the counter-rotating extruders, the polymer essentially does not flow from one screw to the other, as in the co-rotating one. There is a co-rotating movement in the inter-screw gap so that high shear stresses are not produced in this area, as in the co-rotating extrusion.

Counter-rotating extrusion, as with co-rotating extrusion, is usually performed with metered feeding (starve feeding), which results in the pressure profile in the extruder being non-continuous and having sections where the pressure is reduced to zero.

The flow of material in the counter-rotating machine differs totally from that one in the single screw machine but also differs from the one in the co-rotating machine. The essence of this flow is the so-called positive displacement mechanism that does not occur in other extrusion variants. The positive displacement is dependent on the degree of screws meshing. It occurs most fully in the closely intermeshing counter-rotating screws. There is no drag flow here so the energy dissipation is insignificant.

The flow in the counter-rotating machine is mainly performed in a closed space, in the C-shaped chamber, the so-called C-chamber, shown in Figure 2b. There are also various types of leakage flows, schematically shown in Figure 2a.

The C-chamber is bounded by six surfaces, the screw root surface, the barrel surface, the screw flight side surface (twice), and the front surface of the screw flight (twice). There are leakage flows between these surfaces, i.e., the calendering flow Q_c_ in the gap between the screw root and the screw flight, the flight flow Q_f_ in the gap between the screw flight and the barrel, the back pressure inter-screw flow Q_t_ (tetrahedron flow) in the tetrahedral gap between the screw flight flanks (in the radial direction), and the side flow Q_s_ in the side gap between the flight flanks of screws (in the tangential direction).

## 3. Process Modeling

When modeling the twin screw extrusion process, the global approach for modeling is needed. It is necessary to describe the transport of solid polymer, the plasticating of polymer and the flow of molten polymer. The flows of molten polymers are rather well known. However, the transport of solids and polymer plasticating are poorly understood. The correct model of polymer plasticating is fundamental for global process modeling.

### 3.1. Solid Transport

A transport of the solid material in the single screw extruder was modeled first by Darnell and Mol [35] who described the material movement in the screw and the pressure development. They assumed the polymer particles to be compacted into the non-deformable bed, and this solid bed flows as a result of the action of friction forces exerted by the barrel and the screw on the polymer granules. This first, basic model was later extended, e.g., by Tadmor and Broyer [36,37]. The research on solid transport in single screw extruders was reviewed in detail by Schöppner et al. [38,39]. It is worth noting that although many researchers extended the model of Darnell and Mol [35], the principal analysis has not been changed and has been fundamental for the modeling of extrusion for years.

The problem of modeling the transport of solid material in extruders may be solved by using the DEM method (discrete element method). The first studies using this method were performed by Pohl and Potente [40] who modeled the flow in the region of the hopper inflow. The basic extrusion studies were performed by Moysey and Thompson [41,42] who modeled the compacting granules and the pressure development in the screw [43,44]. Advanced modeling of the solid transport using the DEM method was carried out by Schöppner et al. [38,39] who fully modeled the phenomena that occur in the solid transport zone considering the pressure development and the screw filling.

Solid transport in the twin screw extrusion was investigated mostly for co-rotating extruders, for example, by Carrot et al. [45], White and Bawiskar [46], Potente et al. [47], and Wong et al. [48].

The first model of solid transport in the intermeshing co-rotating extruder was developed by Carrot et al. [45]. This model is similar to that commonly used in single-screw extruders, however, it includes two transport mechanisms: the first one in the screw channel, and the second one is the axial transport in the intermeshing area. The model enables to predict the filling of screws towards the geometry and the process operating parameters. 

White and Bawiskar [46] observed that in co-rotating machines there were two areas of solid transport: one in the upper nip between the screws which appeared at the low screw speed and flow rate, and the second one of Archimedean type that was seen near the nip on the underside of the screw and was more important at the high screw speed and flow rate.

Potente and Melish [47] presented a different approach. They divided the conveying zone into the partially filled feed zone and completely filled compression zone. Force and torque balances were drawn up for three different volume elements and, with the solutions obtained for these, it was possible to calculate the maximum conveyable throughput, the pressure profile and the drive power that was required for the solids conveying zone.

Studies on solid transport in counter-rotating machines were limited. Doboczky first [49] discussed this issue, while Wilczyński and White [50] experimentally investigated it. They observed that near the hopper, the solid granules were distributed above the screws and were freely conveyed along the screws. However, the granules were mainly conveyed in the bottom region of the barrel, and only a small fraction of them was conveyed in the upper region of the screws. The granules were collected in the bottom region of the barrel adjacent to the pushing flights of screws, and these subsequently were heated both by the barrel and by being dragged into the gap between the screws.

### 3.2. Plasticating

**Figure 3:**
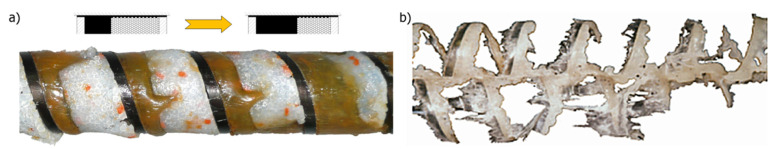
Melting mechanisms for polymer extrusion: (**a**) CSM mechanism (Contiguous Solid Melting) for flood fed single screw extrusion [53] (**b**) melting mechanism for counter-rotating twin screw extrusion [54].

The first studies on plasticating in the single screw machine were carried out by Maddock and Street [51,52]. They proposed the “screw pulling out technique” that involved stopping the extruder, rapidly cooling the barrel and then pulling out the screw of the extruder. The cross-sections of the material removed from the screw were enabled to get to know the plasticating mechanism. It was observed that a melt layer was formed between the barrel and the solid which was scraped off by the transverse flow in the screw and was accumulated near the active flight of the screw. The solid was gradually melted by the heat conducted from the barrel and by viscous dissipation in the melt (Figure 3a).

Tadmor et al. [55,56,57] did similar experimentation and developed the first model of polymer plasticating in the single screw extruder which was fundamental for the theory of extrusion and enabled us to develop the first computer model of this process, that is, the EXTRUD program [58]. The model of plasticating was obtained by considering the profiles of velocity and temperature in the polymer melt film and the profile of temperature in the solid bed. An energy balance was made at the interface solid/melt and the mass balance was performed in the solid and in the melt film. These enabled us to predict the plasticating rate. These studies were later extended by a more detailed description of solid conveying [36,37], and by considering some delay in plasticating [59].

Many other studies were performed by using the “screw pulling out” technique, and they confirmed the Tadmor plasticating model. This model was later extended by other researchers and new models were developed for non-conventional screws. These were recently discussed by Wilczyński et al. [60].

The “screw pulling out technique” is a time-consuming method. Therefore, several other concepts were proposed for studying the plasticating of polymers in extrusion. Liu et al. [61] applied glass windows in the barrel, Noriega et al. [62] used advanced optical methods for observing the plasticating profile, and Wang and Min [63] applied an ultrasound-based system for monitoring the plasticating in the twin screw extruder. Aigner et al. [64] proposed an ultrasonic system for determining the plasticating in the single screw extruder. Yu et al. [65] presented a visualization concept with a transparent barrel equipped with four cameras to observe the flow patterns of polymers in a novel type of co-rotating non-twin screw geometry.

Many researchers extended the models of Tadmor and Klein [5], however, the basic analysis remained unchanged and was usually the basis for modeling the extrusion process.

It is important to note that the plasticating models presented so far, that is the models based on the a priori assumed mechanism are not universal and are not valid for various polymers, various operating conditions, and various screw geometry. These models are useful only for predicting qualitatively the trends in plasticating of polymers in extruders.

Plasticating of polymers in extruders can be simulated by solving the conservation equations of fluid mechanics with a constitutive equation for the polymer being melted.

This concept was presented first by Viriyayuthakorn and Kassahun [66] who developed the 3D FEM (Finite Element Method) model without assuming any plasticating mechanism. The modeling problem was solved with the use of functional dependence of the specific heat on temperature. The solution of equations of motion and energy determined the solid/melt regions which were defined by the temperature distribution. Syrjäla [67] made 2D simulations of plasticating without any mechanism of it assumed. However, in both cases, the simulations were not validated by experiment.

Altinkaynak et al. [68] carried out experimental and theoretical research on the modeling of plasticating using this approach. The two-phase flow of solid and melt was modeled with the Cross-WLF rheological equation which enabled to determine the solid as a high-viscous fluid, while the melt was determined as a low-viscous fluid. Hopmann et al. [69] solved this problem with the use of FVM (Finite Volume Method) method, applying the rheological model of Carreau. Kazmer et al. [70] developed this approach for barrier screws and Lewandowski and Wilczyński [71] for conventional screws.

Contrary to the plasticating in single screw extruders, the research on plasticating in twin screw extruders was limited. This research involved mostly self-wiping co-rotating extruders, e.g., by White and Bawiskar [46], Todd [72], Sakai [73], Wong [74], Carneiro et al. [75,76], and Gogos et al. [77,78,79,80]. Potente and Melish [81], and White and Bawiskar [82] developed the models fundamentally based on the classical Tadmor model [5] for single screw extrusion, assuming the gradual development of a melt layer from the barrel towards the screw. White and Bawiskar [82] modeled the formation of two stratified layers of molten polymer in contact with the hot barrel and solid granules in contact with the colder screw. Potente and Melisch [81] proposed a model based on plasticating the particals uniformly suspended in the molten polymer. Similar model was proposed by Liu et al. [61]. Vergnes et al. [83,84] and Zhu et al. [85] developed the models based on the analysis of the flow of solid/liquid dispersion with some equivalent viscosity. These studies were reviewed and discussed by Teixeira [86].

Plasticating in counter-rotating extruders is less known. Limited observations were presented by Janssen [87], and White et al. [88,89] which indicated that plasticating takes place faster than in intermeshing co-rotating extruders. Wilczyński and White [50] proposed the mechanism of plasticating in intermeshing counter-rotating extruders. They observed that plasticating is initiated both between the screws and at the barrel. The plasticating between the screws is initiated by frictional work on the granules by the calendering stresses between the screws. The plasticating action at the barrel is induced by a barrel temperature higher than the melting point and propagated by viscous dissipation heating of the melt film produced. Based on these observations, the models were developed for plasticating in both those regions [54]. Further studies on plasticating were presented by Wang and Min [63,90] and by Wilczyński et al. [91].

### 3.3. Flow of Molten Polymer

Rowell and Finlayson [92] were the first to analyze the Newtonian flow of viscous oils in single screw machines (screw pumps) and developed the models of drag flow and pressure flow. This analysis was extended to extrusion of polymers by Carley et al. [93] who modeled one-dimensional flow through a rectangular channel of infinite width. Later, the transverse flow caused by the screw flights was considered by Carley and Strub [94], and Squires [95], the effect of channel curvature was investigated by Booy [96] and Squires [97]), as well as the effect of flight clearance was modeled by Mallouk and McKelvey [98], and Maddock [99].

Later studies included the non-Newtonian, one- and two-dimensional, isothermal flow of the power-law fluid in a channel of infinite width, which were discussed and extended by McKelvey [3] and Tadmor and Klein [5].

Since the basic models of Tadmor and Klein [5], many studies were performed to improve the flow modeling in extruders, mostly by describing the two- or three-dimensional flow in the actual screw geometry or by improving the thermal analysis, particularly discussing the mechanical/thermal coupling, e.g., by Syrjäla [100,101], Ilinca and Hetu [102]. Miethlinger et al. [103,104,105] developed a novel heuristic approach for modeling the two- or three-dimensional flow of the power-law fluid in single screw extruders. The issue of modeling the melt flow in single screw extruders was recently reviewed and discussed by Wilczyński et al. [60].

The studies on modeling the melt flow in twin screw extruders were limited. Erdmenger [106,107] was the first to study the flow in co-rotating extrusion. He observed that the polymer moved forward in a roughly helical eight-figure motion. The mechanism of flow in the co-rotating machine is of drag type much as that in the single screw machine. The geometry of the co-rotating screw configuration was discussed in detail by Booy [108]. The Newtonian flow in the fully filled elements was modeled by Booy [109], Denson and Hwang [110], Szydłowski and White [111,112], and Tayeb et al. [113,114]. Later, the non-Newtonian flow was modeled, e.g., by White et al. [115,116,117,118] and Potente et al. [119]. A fully 3D non-Newtonian FEM modeling was made by Ilinca and Hetu [102], Malik et al. [120], and Vergnes et al. [121]. Avalosse and Rubin used the software Polyflow to model co-rotating extrusion [122,123], and recently, Steinbichler et al. [124,125] presented a novel approach to modeling the co-rotating extrusion based on the process parametric study.

Intermeshing counter-rotating extruders fundamentally differ from the single screw machines, and from the co-rotating machines. Kiesskalt [126], Montelius [127] and Schenkel [2] were the first to consider these machines as positive displacement pumps. Doboczky [49,128] and Janssen [87,129] developed the pumping characteristics for these machines, and they first discussed the leakage flows. White and Adewale [130] modeled the flow considering the level of intermeshing in the machine. Numerical FEM simulations presented Li and Manas-Zloczower [131], and Kajiwara et al. [132]. Hong and White [133,134] used a FAN (Flow Analysis Network) analysis to model the non-Newtonian flow. They developed screw pumping characteristics for various screw elements which allowed the modelling of the flow in various screw designs and calculate the pressure, fill factor, and temperature profiles. Shah and Gupta [135,136] compared the flow in co-rotating and counter-rotating machines. Recently, Wilczyński and Lewandowski [137] presented the fully 3D non-Newtonian FEM computations to develop the pumping characteristics. These computations included the flow in the C-chamber and the leakage flows.

Nowadays, 3D FEM simulations are available for single screw and twin-screw extrusion which allow the description of the velocity and temperature distributions and the pressure/flow rate relations, however, they require large computing resources and major calculation time. Therefore, these computations cannot be applied for global process modeling which requires hundreds of computing iterations. To avoid the time-consuming computations, the concept of screw pumping characteristics was developed which are defined by the dimensionless flow rate and the dimensionless pressure gradient [9]. These characteristics can be described by the regression models and implemented into the iterative procedures with reasonable computation accuracy and computation time. They were developed both for single screw and twin-screw extruders, e.g., by White and Potente [9], Rauwendaal [13], and recently by Wilczyński et al. [138,139,140].

## 4. CFD Modeling of Twin Screw Extrusion

In general, the global models which describe the complete extrusion process including solid transport, polymer melting and melt flow are the lumped parameter models, and are based on the relatively simple 1D or 2D models, and do not allow looking into details of the process. CFD modeling allows studying the process in detail calculating e.g., distributions of shear stress, shear rate, viscosity, residence time, etc.

Three-dimensional FEM computations with the use of CFD (Computational Fluid Dynamic) software are available for both, single screw and twin screw extruders. These allow the description of the velocity and temperature distributions and the pressure/flow rate relations, and many others. The CFD software is a powerful tool for modeling of polymer extrusion, however, its use is not easy. It requires a good knowledge of the process under study, a good understanding of the modeling procedures, and correct formulating of the boundary conditions, as well as proper interpretations of the modeling results. These issues were discussed in the recently published book of Wilczyński [34].

ANSYS Polyflow software [141] can be used for modeling twin screw extrusion. It is the FEM computational fluid dynamics (CFD) program for simulating the viscous and viscoelastic flows which can be isothermal or non-isothermal, two- or three-dimensional, steady-state or time-dependent. It is primarily used for solving the flow problems in polymer processing; however, it can be also used for solving the rheology problems of other materials such as glass or foodstuffs.

The flow of polymer in co-rotating extruders is the result of the relative movement of the screws and the barrel and the pressure gradient in the machine. It is the pressure-drag flow which takes place in open channels between screws (alternately one and the other) and the barrel (Figure 1). In co-rotating extruders, during each screw rotation, the polymer is transferred from one screw to the other, and moves in an open figure-eight pattern, along the so-called twisted eight. The flow of polymer is dragged, so that it resembles the flow in a single screw extruder in this respect. However, when moving between the screws, the polymer travels a longer path and undergoes higher shearing. There is a counter-rotating movement in the inter-screw gap, as a result of which high shear stresses are produced in this area.

The flow of polymer in a counter-rotating extruder totally differs from that one in a single screw extruder, but also differs from that one in a co-rotating extruder. The essence of this flow is the so-called positive displacement mechanism that does not occur in other extrusion processes. The degree of positive displacement is dependent on the degree of screw meshing. It occurs most fully in the closely intermeshing counter-rotating extruders. There is no drag flow so that the energy dissipation is insignificant. The flow in a counter-rotating machine is mainly performed in a closed space, in the C-shaped chamber, shown in Figure 2b. Since the channels are closed here, the material does not flow from one screw to the other except for the leakage flows, schematically shown in Figure 2a, which reduce the degree of positive conveying. There is a co-rotating movement in the inter-screw gap so that high shear stresses are not produced in this area, as in the case of co-rotating extrusion.

In twin screw extrusion, both co-rotating and counter-rotating, the flow is three-dimensional and unsteady since the screws rotate. To simplify the set-up of modeling of such a flow problem, the mesh superposition technique (MST) has been developed for the Polyflow software [141]. A finite element mesh is built for each part of the flow: one for the flow domain (Subdomain 1) that is the inner part of the barrel without the screws, and one for each screw (Subdomain 2, Subdomain 3). The set-up of the flow problem consists in defining the boundary conditions. The screws rotate at a given screw speed N in the same directions (co-rotating) or in the opposite direction (counter-rotating), and the velocity vanishes at the barrel. The flow rate is imposed at the entry to the computational domain, and it is also the outflow condition at the exit of the domain. In this paper, the flow problem has been defined as an isothermal, 3D generalized Newtonian flow of the power law fluid.

**Figure 4:**
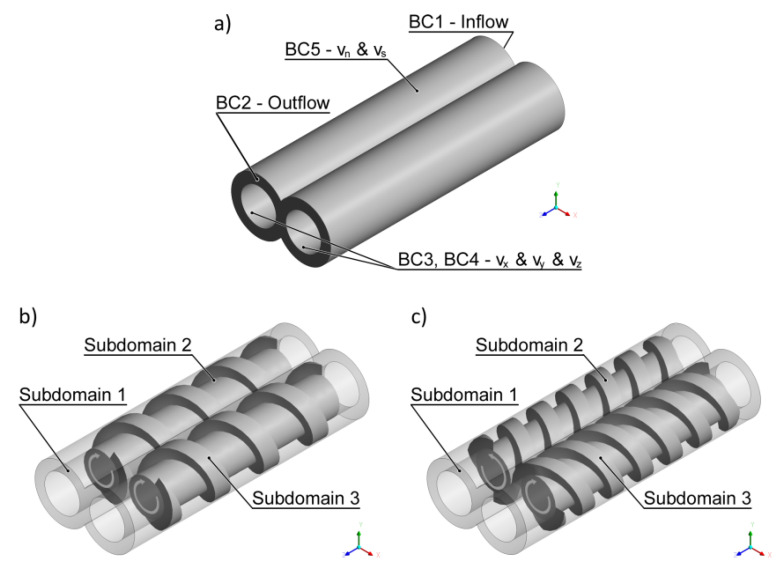
The flow geometry and boundary conditions for twin screw extrusion: (**a**) boundary conditions: BC1—inflow, BC2—outflow, BC3/BC4—Cartesian velocity, BC5—zero wall velocity, (**b**) co-rotating extrusion, (**c**) counter-rotating extrusion (adopted with permission from: Wilczyński, K. *Rheology in Polymer Processing. Modeling and Simulation*; Carl Hanser Verlag: Munich 2021 [34]).

The computation scheme is depicted in Figure 4. Three subdomains are distinguished, for the flowing material (Subdomain 1) and for the screws (Subdomain 2, Subdomain 3).

The following flow boundary conditions were applied to define the process:

− at the inlet to the flow domain: boundary BC1—inflow, the flow rate (Q_in_) = (Q) is imposed,
− at the outlet of the flow domain: boundary BC2—*outflow*, vanishing normal forces and tangential velocities are imposed (f_n_ and v_s_) = (0, 0), which means that the flow rate (Q_out_) = (Q) is imposed,
− on the screw domain: boundary BC3—*Cartesian velocity*, Cartesian velocities are imposed (v_x_ and v_y_ and v_z_) = (–N)—for co-rotating extrusion, which means that the screw is rotated at the screw speed (N) counterclockwise, or (v_x_ and v_y_ and v_z_) = (N)—for counter-rotating extrusion, which means that the screw is rotated clockwise,
− on the screw domain: boundary BC4—*Cartesian velocity*, Cartesian velocities are imposed (v_x_ and v_y_ and v_z_) = (–N) for both co-rotating and counter-rotating extrusion, which means that the screw is rotated at the screw speed (N) counterclockwise,
− on the barrel wall: boundary BC5—*zero wall velocity*, vanishing normal and tangential velocities are imposed (v_n_ and v_s_) = (0, 0), which means that the velocity vanishes at the barrel wall.

The boundary conditions, BC1—inflow (Q_in_) = (Q), and BC2—*outflow*, i.e., normal forces and tangential velocities (f_n_ and v_s_) = (0, 0), imply that pressure may be developed over the screws. Since the pressure at the screw element end is not known, the pressure gradient is computed relative to the zero pressure at the element exit. Negative pressures which may result from simulations do not mean negative pressures in the extrusion process.

The simulations were performed to study the flow parameters distributions for different pressure gradients: positive gradient (∂p/∂z > 0, i. e., Δp < 0), zero gradient (∂p/∂z = 0, i. e., Δp = 0), and negative gradient (∂p/∂z < 0, i. e., Δp > 0), where Δp is the pressure difference between the pressure at the channel inlet and the pressure at the channel outlet.

In the case of co-rotating extrusion, the simulations started with the initial boundary condition BC1_initial_—*outflow* (Q_out_) = (Q), with other conditions unchanged, which implies that pressure is not generated along the screw, and the flow rate is computed which is equal to the drag flow Q_d_, that is, there is no pressure flow, and we have (∂p/∂z = 0, i. e., Δp = 0). Then, the simulations were performed with the basic boundary condition BC1—inflow (Q_in_) = (Q), for different values Q = 0, Q = Q_d_, Q = 2Q_d_, which corresponds to the different pressure gradients: positive gradient, zero gradient, and negative gradient.

In the case of counter-rotating extrusion, the simulations started with the initial boundary condition BC1_initial_—outflow (Q_out_) = (Q), with other conditions unchanged, which implies that pressure is not generated along the screw, and the flow rate is computed which is equal to the net flow Q_net_, that is, there is no pressure flow, and we have (∂p/∂z = 0, i. e., Δp = 0). The net flow is composed of the chamber flow and the drag components of leakage flows. Then, the simulations were performed with the basic boundary condition BC1—inflow (Q_in_) = (Q), for different values Q = 0, Q = Q_net_, Q = 2Q_net_ which corresponds to the different pressure gradients: positive gradient, zero gradient, and negative gradient.

**Figure 5:**
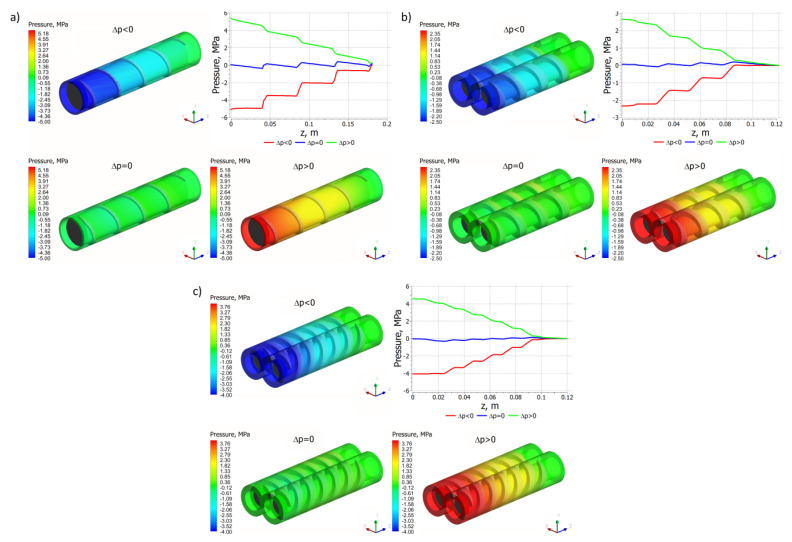
Pressure distributions for extrusion at various pressure gradients (∂p/∂z), positive (Δp < 0), zero (Δp = 0) and negative (Δp > 0): (**a**) single screw extrusion, (**b**) co-rotating twin screw extrusion, (**c**) counter-rotating twin screw extrusion (adopted with permission from: Wilczyński, K. *Rheology in Polymer Processing. Modeling and Simulation*; Carl Hanser Verlag: Munich 2021 [34]).

Pressure distributions are depicted in Figure 5, which in general are similar each other, although they differ in detail. In the case of single screw extrusion (Figure 5a) and co-rotating extrusion (Figure 5b), for the positive pressure gradient, the pressure flow is subtracted from the drag flow, while for the negative gradient, the pressure flow is added to the drag flow. In the case of the zero-pressure gradient, the pressure flow does not appear. In the case of counter-rotating extrusion (Figure 5c), for the positive pressure gradient, the pressure leakage flows decrease the flow rate, while for the negative gradient these increase the flow rate. In the case of the zero-pressure gradient, the pressure leakage flows do not appear. However, the drag components of the leakage flows still exist.

It is worth noting the characteristic pressure pulsation for all the screw configurations (single, co-, and counter-), the so-called pressure saw, which is the result of varying screw geometry in a given area of an analysis. At a certain point in the flow space, the screw flight and the screw channel alternate, which generates pressure pulsation. This phenomenon is an inherent and always occurring feature of screw flows. It is also the cause of pulsation in the extruder output. The frequency of this pulsation is equal to the frequency of screw rotation. Counter-rotating twin screw extrusion seems to be the most stable process, the pulsations are much lower than for single screw extrusion or co-rotating extrusion.

**Figure 6:**
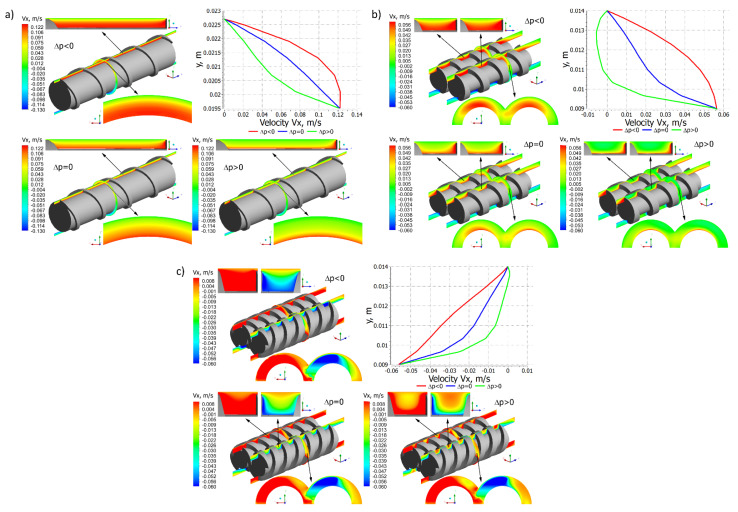
Distributions of the velocity v_x_ for extrusion at various pressure gradients (∂p/∂z), positive (Δp < 0), zero (Δp = 0) and negative (Δp > 0): (**a**) single screw extrusion, (**b**) co-rotating twin screw extrusion, (**c**) counter-rotating twin screw extrusion (adopted with permission from: Wilczyński, K. *Rheology in Polymer Processing. Modeling and Simulation*; Carl Hanser Verlag: Munich 2021 [139]).

**Figure 7:**
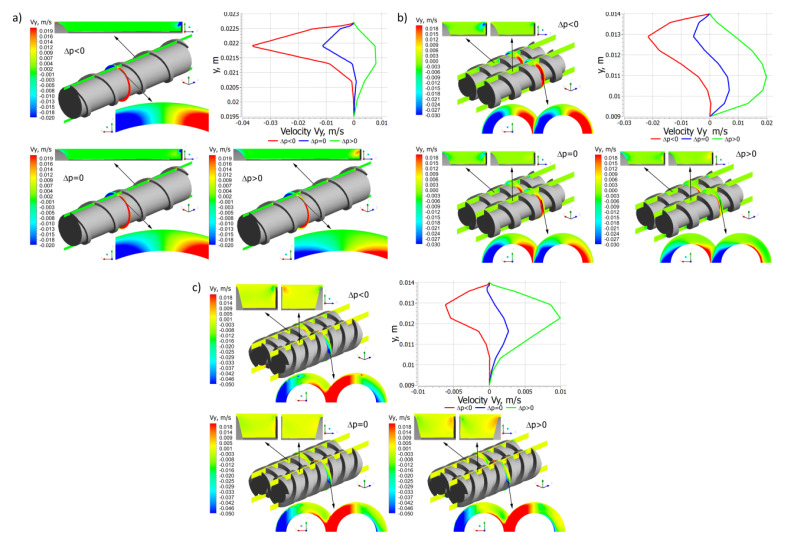
Distributions of the velocity v_y_ for extrusion at various pressure gradients (∂p/∂z), positive (Δp < 0), zero (Δp = 0) and negative (Δp > 0): (**a**) single screw extrusion, (**b**) co-rotating twin screw extrusion, (**c**) counter-rotating twin screw extrusion (adopted with permission from: Wilczyński, K. *Rheology in Polymer Processing. Modeling and Simulation*; Carl Hanser Verlag: Munich 2021 [34]).

**Figure 8:**
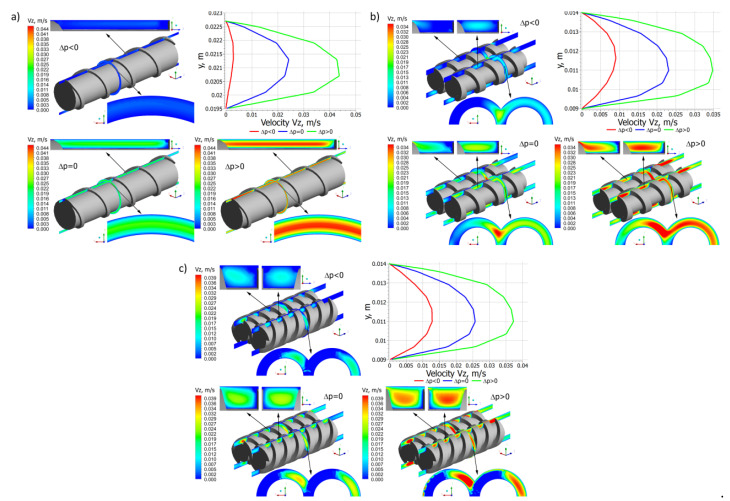
Distributions of the velocity v_z_ for extrusion at various pressure gradients (∂p/∂z), positive (Δp < 0), zero (Δp = 0) and negative (Δp > 0): (**a**) single screw extrusion, (**b**) co-rotating twin screw extrusion, (**c**) counter-rotating twin screw extrusion (adopted with permission from: Wilczyński, K. *Rheology in Polymer Processing. Modeling and Simulation*; Carl Hanser Verlag: Munich 2021 [34]).

Distributions of the velocity components for different pressure gradients are shown in Figure 6, Figure 7 and Figure 8. In general, in the case of single screw extrusion and co-rotating extrusion, these are similar, and totally different than in the counter-rotating extrusion.

For single screw extrusion, the velocity component v_x_ (Figure 6a), which is perpendicular to the screw axis, is decreased by the pressure flow at the positive pressure gradient and is increased by the pressure flow at the negative gradient. The velocity component v_y_ (Figure 7a), also perpendicular to the screw axis, varies depending on the pressure gradient. The velocity component v_z_ (Figure 8a), which is parallel to screw axis, has a parabolic distribution, and increases with an increase of the pressure difference Δp.

For co-rotating extrusion, the velocity component v_x_ (Figure 6b) is similarly decreased by the pressure flow at the positive pressure gradient and is increased by the pressure flow at the negative gradient. It is worth noting that distributions are almost the same in both screws. The velocity component v_y_ (Figure 7b) varies depending on the pressure gradient. The velocity component v_z_ (Figure 8b) has a parabolic distribution and increases with an increase of the pressure difference Δp. These distributions are similar but not the same in both screws.

For counter-rotating extrusion, the velocity component v_x_ (Figure 6c) is decreased at the positive pressure gradient and is increased at the negative gradient. It is worth noting that distributions are totally different in both screws. The velocity component v_y_ (Figure 7c) varies depending on the pressure gradient. The velocity component v_z_ (Figure 8c) has a parabolic distribution and increases with an increase of the pressure difference Δp. These distributions are similar in both screws but not the same.

**Figure 9:**
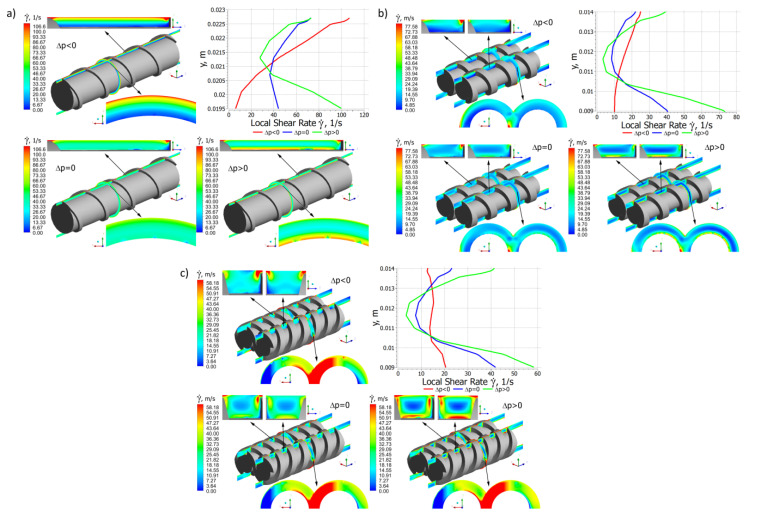
Distributions of the shear rate for extrusion at various pressure gradients (∂p/∂z), positive (Δp < 0), zero (Δp = 0) and negative (Δp > 0): (**a**) single screw extrusion, (**b**) co-rotating twin screw extrusion, (**c**) counter-rotating twin screw extrusion (adopted with permission from: Wilczyński, K. *Rheology in Polymer Processing. Modeling and Simulation*; Carl Hanser Verlag: Munich 2021 [34]).

**Figure 10:**
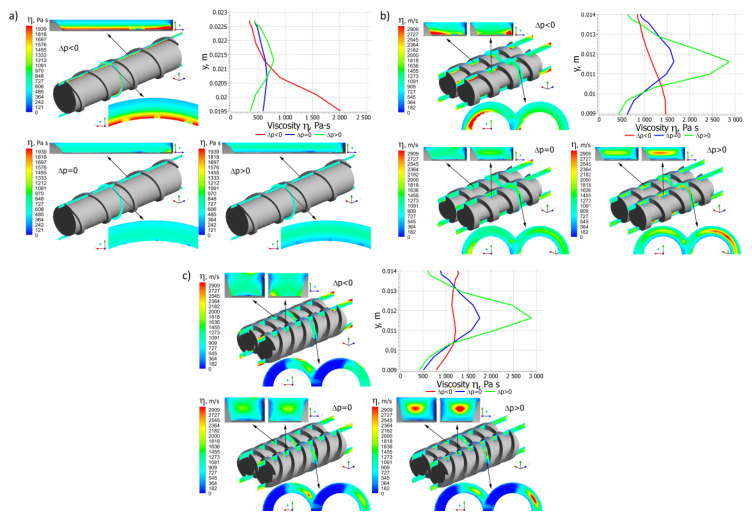
Distributions of the viscosity for extrusion at various pressure gradients (∂p/∂z), positive (Δp < 0), zero (Δp = 0) and negative (Δp > 0): (**a**) single screw extrusion, (**b**) co-rotating twin screw extrusion, (**c**) counter-rotating twin screw extrusion (adopted with permission from: Wilczyński, K. *Rheology in Polymer Processing. Modeling and Simulation*; Carl Hanser Verlag: Munich 2021 [34]).

Distributions of the local shear rate and the local viscosity for different pressure gradients are presented in Figure 9 and Figure 10.

In the case of single screw extrusion (Figure 9a and Figure 10a), for the positive pressure gradient, the shear rate increases from the screw to the barrel, while for the negative and zero gradients it has a minimum in the middle of the channel. These correspond to the velocity distributions v_x_, v_y_, and v_z_ (Figure 6a, Figure 7a and Figure 8a). The viscosity distribution results from the shear rate distribution. For the positive pressure gradient, it decreases from the screw to the barrel, while for the negative and zero gradients it has a maximum in the middle of the channel.

In the case of co-rotating extrusion (Figure 9b and Figure 10b), for the positive pressure gradient, the shear rate increases from the screw to the barrel, while for the negative and zero gradients it is highest at the screw and barrel surfaces with a minimum in the middle of the channel. These correspond to the velocity distributions v_x_, v_y_, and v_z_ (Figure 6b, Figure 7b and Figure 8b). For the positive pressure gradient, the viscosity decreases from the screw to the barrel, while for the negative and zero gradients it is lowest at the screw and barrel surfaces with a maximum in the middle of the channel.

In the case of counter-rotating extrusion (Figure 9c and Figure 10c), for the positive pressure gradient, the shear rate decreases from the screw to the barrel, while for the negative and zero gradients it is highest at the screw and barrel surfaces with a minimum in the middle of the channel. These correspond to the velocity distributions v_x_, v_y_, and v_z_ (Figure 6c, Figure 7c and Figure 8c). For the positive pressure gradient, the viscosity increases from the screw to the barrel, while for the negative and zero gradients it is lowest at the screw and barrel surfaces with a maximum in the middle of the channel.

**Figure 11:**
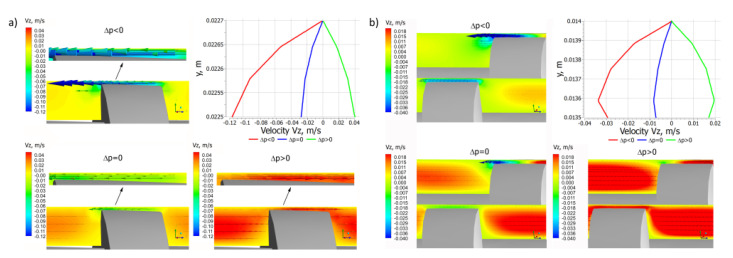
Distributions of the velocity v_z_ for the leakage flow in extrusion at various pressure gradients (∂p/∂z), positive (Δp < 0), zero (Δp = 0) and negative (Δp > 0): (**a**) single screw extrusion, (**b**) co-rotating twin screw extrusion (adopted with permission from: Wilczyński, K. *Rheology in Polymer Processing. Modeling and Simulation*; Carl Hanser Verlag: Munich 2021 [34]).

The flow of polymer in a co-rotating twin screw extruder is similar to some extent to the flow in a single screw extruder. This is the pressure-drag flow with the leakage flow in the gap between the tips of the screws and barrel, which affects the flow rate. The leakage flows in the single screw extruder and in the co-rotating extruder are depicted in Figure 11 for different pressure gradients. These flows are the pressure-drag flows. For the positive pressure gradient, the pressure leakage flow decreases the flow rate, while for the negative gradient, it increases the flow rate. In the case of the zero-pressure gradient, the pressure leakage flow does not appear. However, the drag component of the leakage flow still exists. It is worth noting that the viscosity of polymer in the gap is much lower than the viscosity of polymer in the channel, since the shear rate in the gap is much higher than in the screw channel.

The flow in a counter-rotating extruder is mainly performed in the C-chamber by positive displacement. However, there are also various leakage flows (Figure 2) which reduce the degree of positive conveying and affect the flow rate.

The following leakage flows can be distinguished:

− the calendering flow Q_c_ in the gap between the screw root and the screw flight,
− the flight flow Q_f_ in the gap between the screw flight and the barrel,
− the back pressure inter-screw flow Q_t_ (tetrahedron flow) in the tetrahedral gap between the screw flight flanks, in the radial direction,
− the side flow Q_s_ in the gap between the screw flight flanks, in the tangential direction.

**Figure 12:**
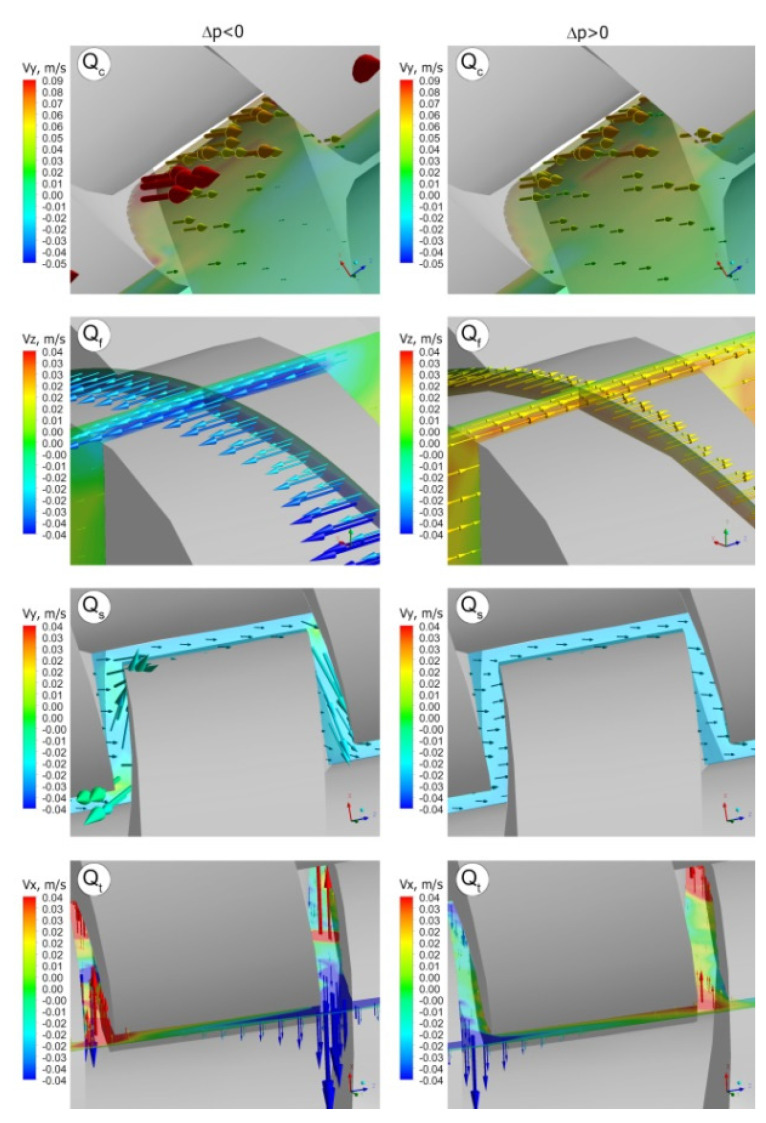
Leakage flows in the counter-rotating twin screw extrusion for different pressure gradients (∂p/∂z), positive (Δp < 0) and negative (Δp > 0): Q_c_—calendering leakage flow, Q_f_—flight leakage flow, Q_s_—side leakage flow, Q_t_—pressure leakage flow (adopted with permission from: Wilczyński, K. Rheology in Polymer Processing. Modeling and Simulation; Carl Hanser Verlag: Munich 2021 [34]).

The leakage flows are pressure-drag flows, except the tetrahedral flow which is a pressure flow. The total leakage flow may be presented as a sum of drag flows and pressure flows. The leakage flows at the positive and negative pressure gradients are shown in Figure 12. At the positive gradient the leakage flows are more intense. Moreover, the flight flow and the tetrahedron flow change the direction when the sign of pressure gradient changes.

**Figure 13:**
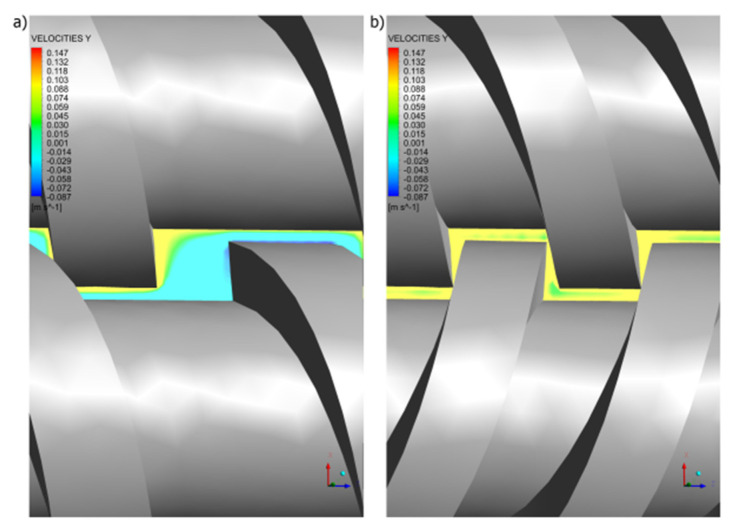
Distributions of the velocity v_y_ in the gap between screw flights and screw root: (**a**) co-rotating extrusion, (**b**) counter-rotating extrusion.

**Figure 14:**
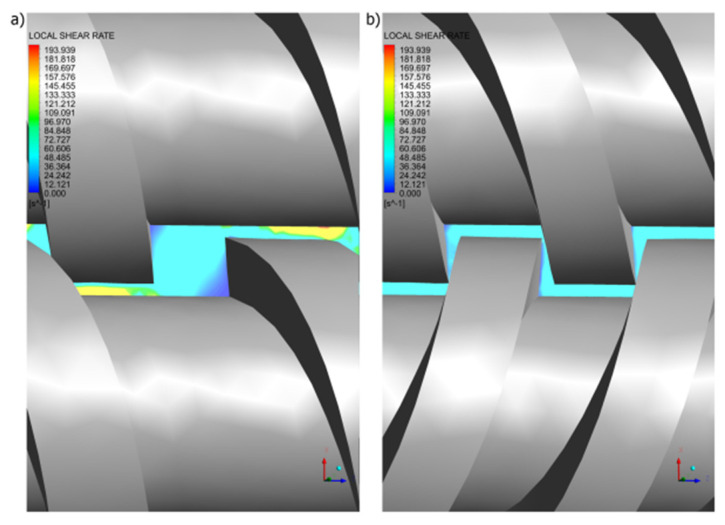
Distributions of the shear rate in the gap between screw flights and screw root: (**a**) co-rotating extrusion, (**b**) counter-rotating extrusion.

It is interesting to note that in the co-rotating extrusion, there is a counter-rotating movement in the inter-screw gap (Figure 13a), as a result of which high shear rates are produced in this area (Figure 14a). However, in the counter-rotating extrusion, there is a co-rotating movement in the inter-screw gap (Figure 13b), so that high shear rates are not produced in this area (Figure 14b), as in the co-rotating extrusion.

## 5. Computer Models of Twin Screw Extrusion

The issue of modeling of polymer extrusion was discussed in books, by White and Potente [9], Rauwendaal [13], Agassant et al. [27], and was reviewed in papers, by Arrifin et al. [142], Wilczyński et al. [143], Teixeira et al. [144], Malik et al. [120], and recently by Hyvärinen et al. [145] and Wilczyński et al. [60]. Several specific issues of twin screw extrusion were discussed by Vergnes [146], Vlachopoulos et al. [147], and Bauera et al. [148]. Dhaval et al. [149], Lee and Kim [150], and Ravikumar et al. [151] presented review papers on applications of twin screw extrusion outside the polymer processing industry.

The first computer program for simulating the extrusion process EXTRUD was developed by Tadmor and Klein [57] which was later extended by Klein and Klein [152,153] as the SPR extrusion simulating program. Next, several other systems were built, by Vlachopoulos and Agur [153], Vincelette et al. [154], Potente et al. [155,156], Sebastian and Rakos [157], Amellal and Lafleur [158], and Wilczyński [159,160,161]. These were recently discussed in [60].

Studies on co-rotating extrusion were started by White group. Using the melt flow models [117,118] and the plasticating models [46,82], they developed the Akro-Co-Twin program [162,163,164]. Potente et al. based on the process studies [47,81,119] developed the SIGMA system [165,166]. Vergnes et al. based on their process studies [83,84,114] built the LUDOVIC system [167]. Canedo [168] developed the TXSTM program, and Teixeira et al. [145] built the global modeling system for co-rotating extruders.

Studies on counter-rotating extrusion were also started by the white group. Using the melt flow models [134,135] and the plasticating models [50,54], they developed the Akro-Counter-Twin program [169,170]. This research was continued by Wilczyński et al. [171,172] who built the TSEM system (Twin Screw Extrusion Model).

At present, the most important software for twin screw extrusion is Ludovic [173] and Sigma [174]. This software makes it possible to predict the thermo-mechanical process parameters (temperature, pressure, melting, viscosity, filling) along the screw profile, detailed energy balance of the process, residence time analysis, and many others. These handle all the process parameters for a simulation: the geometry/screw design, the material characteristics, the process conditions. Performing a Design of Experiments (DOE) allows us to study the impact of input parameters variation (defined by the user) on the material/process outputs. Performing a DOE means hundreds of simulations which provide trends on the material reaction to hundreds of potential process configurations. Scaling the process is possible to transfer the process to extruders larger or smaller diameters. Starting from the so-called model process, the geometry of the target design and the associated process parameters are calculated on the basis of transfer rules. The starting point for the scale-up is any billable process.

Ludowic and Sigma allow for simulating extrusion of “pure” polymers, as well as extrusion of polyblends and filled polymers. Ludovic can also simulate extrusion of foodstuffs, pharmaceutics and cosmetics, explosives and building materials.

Some materials, e.g., filled polymers and polymer suspensions, exhibit wall slip when processing. This phenomenon was studied first by Mooney [175], and later was reviewed by Potente et al. [176]. Recently, Wilczynski et al. discussed this issue in detail [60]. Lewandowski and Wilczyński [177] presented 3D non-Newtonian FEM studies on the melt flow with slip effects in single screw extrusion to develop the process pumping characteristics to be implemented into the global model of the process. The flow with slip effects both in the screw and in the die was analyzed. Kalyon et al. [178] and Malik et al. [120] investigated numerically slip effects in co-rotating extrusion.

Some materials, e.g., filled polymers, exhibit a yield stress. This phenomenon was discussed first by Bingham [179], and was reviewed by Bird et al. [180], and Mitsoulis [181]. Studies on viscoplastic flows in extrusion were limited. Laval and Kalyon [182,183] first modeled analytically the single screw extrusion of the Herschel–Bulkley viscoplastic fluid. Recently, Wilczyński et al. discussed this issue in detail [60]. Lewandowski and Wilczyński [184] presented 3D non-Newtonian FEM studies on the viscoplastic flows in single screw extrusion to develop the pumping characteristics to be implemented into the global model of the process. The flow with yield stress effects both in the screw and in the die was analyzed. Kalyon et al. [178] investigated experimentally and numerically the flow and heat transfer in twin screw extrusion using the Herschel–Bulkley model.

The essence of extrusion is the co-operation of the screw and the die. The global modeling means the modeling of this co-operation. When global modeling the process, the specific computation algorithm is necessary. For classical extrusion with flood feeding, the forward algorithm is suitable. For extrusion with starvation, the backward (inverse) algorithm is necessary. These issues were discussed in detail in [60].

Modeling algorithms for flood fed single screw extrusion are known [153,159,160]. The modeling is proceeded from the hopper to the die, and the process operating point is searched which is defined by the extrusion throughput and pressure. The flow rate is not known here, the computations start for some initially assumed flow rate, e.g., the drag flow rate, and the process is simulated (solid transport, plasticating, melt flow). The pressure at the die exit is compared to the atmospheric pressure, and the convergence of these is searched.

Modeling algorithms for starve fed extrusion are less known. The modeling requires an inverse approach. The modeling is proceeded from the die to the hopper. The flow rate is known here, and the die pressure is calculated first for some initially assumed temperature. Then, the process is simulated back along the screw using the screw pumping characteristics. When the pressure falls to zero, the starvation starts, and the screw filling is calculated.

This inverse algorithm of computation was used by the authors to develop the global model of counter-rotating extrusion [169,171,172]. Other researchers used this approach for co-rotating extrusion [162,166,167]. These computations are relatively simple in execution since one-stage plasticating models are used here. Moreover, when modeling the co-rotating extrusion, the location of plasticating region is not computed but a-priori specified.

Inverse computations for single screw extrusion with starvation are more complex. The authors, using the polymer plasticating models [53,185], built the first computer model of this process SSEM-Starve [139]. Later, non-conventional screw configurations were taken into account [34,140], and extrusion of polymer blends [186] and composites [187]. Recently, the novel approach to process modeling was presented which allows to model the single screw extrusion both starve fed and flood fed [34]. In these computations the two-stage plasticating models are used, and the location of the plasticating region is not specified a-priori.

## 6. Future Concepts

In extrusion, when modeling the polymer plasticating, the experiment is first performed to get to know the plasticating mechanism, then the physical model of this mechanism is proposed, and finally the mathematical model is developed. Thus, these models are not general in nature and are limited to the specific process and are dependent on the operating and geometrical parameters of the process.

The concept of solving this problem using CFD computations seems to be promising. Instead of a priori assumed the specific solid/melt flow mechanism, the flow of the material in the extrusion can be described by solving the conservation equations (motion and energy).

In the case of starve fed extrusion this concept may be difficult to implement because the flow area (in the partially filled section of the screw) is not defined. However, it seems to be reasonable to evaluate this area using the degree of filling the screw channel with material.

The promising concept would be the coupled CFD/DEM modeling. Recently, such a coupling the DEM software EDEM and the open-sourced CFD software OpenFOAM has been developed.

Optimization and scaling the polymer extrusion are the important tools of process designing. Optimization consists in searching the extreme values in the multidimensional space of the process output parameters. Scaling is about changing the scale of the process. It consists in minimizing the differences between the reference process and the novel process. This may be obtained with the use of optimization techniques leading to the minimization of these differences.

Various optimization techniques have been used for optimization and scaling the polymer extrusion, among them the Genetic Algorithms [188] which have been identified as powerful and very efficient. Covas and Gaspar-Cunha first used the Genetic Algorithms for polymer extrusion and developed optimization procedures for single screw extrusion [189,190,191,192] and co-rotating twin screw extrusion [193,194,195,196,197], as well as developed scaling-up procedures for both single screw extrusion and co-rotating twin screw extrusion [198,199,200]. Recently, Nastaj and Wilczyński [201] broadly reviewed and discussed the issue of optimization and scaling-up the extrusion process. They concluded that the counter-rotating extrusion process has not yet been discussed from the point of view optimization and scaling-up.

## 7. Conclusions

An issue of modeling of twin screw extrusion of polymeric materials has been reviewed. Global modeling has been discussed which includes modeling of solid transport, polymer plasticating, and flow of molten polymer, as well as the screw/die co-operation. Methodology of CFD modeling of twin screw extrusion has been discussed as well as the examples of this modeling has been presented to show the details of this process. The future prospective of developments and research of twin screw extrusion have been presented.

Limitations of the conventional approach to modeling the polymer plasticating based on the assumed polymer solid/melt flow mechanism were discussed, and the concept of modeling by solving the conservation equations (motion and energy) without any assumed mechanism of polymer flow has been presented. Attention has been paid to the promising progress in extrusion modeling with the use of the coupled DEM/CFD computing procedures.

## Data Availability

The data presented in this study are available on request from the corresponding author.
